# Aneuploidy: a common and early evidence-based biomarker for carcinogens and reproductive toxicants

**DOI:** 10.1186/s12940-016-0180-6

**Published:** 2016-10-12

**Authors:** Daniele Mandrioli, Fiorella Belpoggi, Ellen K. Silbergeld, Melissa J. Perry

**Affiliations:** 1 Cesare Maltoni Cancer Research Center, Ramazzini Institute, 40010 Bentivoglio, Bologna, Italy; 2 Department of Environmental Health Sciences, Johns Hopkins Bloomberg School of Public Health, 21205 Baltimore, MD USA; 3 Department of Environmental and Occupational Health, Milken Institute School of Public Health, The George Washington University, 950 New Hampshire Ave. NW, 4th Floor, Washington, DC 20052 USA

**Keywords:** Aneuploidy, Carcinogens, Reproductive toxicants, Endocrine disruptors, Chromosomes, Early diagnose, Bioassays, Risk assessment, mFISH, Laser scanning microscopy

## Abstract

Aneuploidy, defined as structural and numerical aberrations of chromosomes, continues to draw attention as an informative effect biomarker for carcinogens and male reproductive toxicants. It has been well documented that aneuploidy is a hallmark of cancer. Aneuploidies in oocytes and spermatozoa contribute to infertility, pregnancy loss and a number of congenital abnormalities, and sperm aneuploidy is associated with testicular cancer. It is striking that several carcinogens induce aneuploidy in somatic cells, and also adversely affect the chromosome compliment of germ cells. In this paper we review 1) the contributions of aneuploidy to cancer, infertility, and developmental abnormalities; 2) techniques for assessing aneuploidy in precancerous and malignant lesions and in sperm; and 3) the utility of aneuploidy as a biomarker for integrated chemical assessments of carcinogenicity, and reproductive and developmental toxicity.

## Background

Biomarker of effect is defined as a measurable biochemical, physiologic or other alteration in an organism that is associated with an established or possible health impairment or disease [1]. Effect biomarkers have a central role for toxicological evaluations including in vitro studies in tissue samples, in vivo studies in animal models, and for early disease detection and monitoring of health status in humans [2, 3]. Biomarkers of effect are widely explored in medicine [4], environmental epidemiology [5] and public health [6]. Together with the benefits for health and safety, using effect biomarkers may result in lower costs to the public, industry and governments favoring cost-effective approaches for disease prevention [7]. They can contribute to early detection and prediction of adverse effects, supporting prioritization and screening programs for substances of concern [8]. Their utility has resulted in an increasing demand for new and informative biomarkers of effect. Aneuploidy, because its mechanisms are well known and its adverse effects are well established, is being increasingly used as an informative effect biomarker [9, 10]. Aneuploidy is a hallmark of cancer and causes developmental abnormalities in all species where the condition has been examined [11, 12]. Aneuploidy is defined as “one or more whole chromosomes (numerical chromosomal aneuploidy) absent from or in addition to the euploid complement, or having one or more chromosome segments (segmental or structural aneuploidy) absent from or in addition to the euploid complement” [13]. The euploid complement, or the normal content of chromosomes for a cell, always exists as an exact multiple of the haploid number of chromosomes: germinal cells are haploid (1 copy of each chromosome, 23 chromosomes total), somatic cells are generally diploid (2 copies of each chromosome, 46 chromosomes total) and in some rare cases can be poliploid (more than 2 copies of each chromosome, for example muscle, macrophages). Therefore any gain or loss of chromosomal material by the euploid complement creates an-euploidy (in Greek euploidy literally means “normal form” –of the nucleus-, while an-euploidy means “abnormal form”). The role of aneuploidy in cancer and reproductive biology has been investigated since the last century, but only relatively recently have technical advances translated early findings from basic science to experimental, pathological and epidemiological settings. Following the early pioneering works by David Hansemann [14], it was the talented couple Theodore Boveri and Marcella O’Grady [15, 16] that in 1904 first provided systematic and detailed evidence of the role of aneuploidy in fertilization, development and cancer [17–20]. They were already aware that chemical and occupational exposures could induce cancer, another pioneering view for the time, “the connection between cancer and certain chemical irritants is even clearer than it is between cancer and the physical agents I have mentioned. I need only refer to the cancers of paraffin workers.” [21]. Other historical steps that elucidated the role of aneuploidy in cancer, reproductive, and developmental toxicity include: 1929, Barbara McIntock discovers the disruptive role of aneuploidy in maize development [22]; 1956, Julian Huxley introduces the concept of cancer as a new biological species with a high degree of genetic heterogeneity [23]; 1959, Jerome Lejeun discovers that an extra copy of Chromosome 21 causes Down Syndrome [24]; 1961, Theodore Hauschka documents that the euploid content in human and rodent tissues is strongly conserved and observes that leukemia cells are consistently aneuploid [25]; 1971, Andreĭ Pavlovich Dyban and Vladislav Sergeevitch Baranov show through Robertsonian translocation the reproductive and developmental effects of all the possible trisomies in mice [26]; and 1986, Mitsuo Oshimura and J. Carl Barrett elucidate the mechanisms of chemical induced aneuploidy in mammalian cells [27].

The mechanisms that link aneuploidy to adverse outcomes are well determined: aneuploid germinal cells encounter difficulty in normal fertilization and development and aneuploid tumor cells never express a normal phenotype. Down Syndrome is the best example of how a single extra copy of one of the smallest chromosomes (containing normal genes) may disrupt organs and cellular phenotypes. Down Syndrome patients with complete trisomy 21 present more severe symptoms and a more disrupted phenotype than incomplete trisomy 21 [28–30]. In humans, copy number changes of autosomes (non-sex chromosomes containing normal genes) are generally not compatible with normal development or survival. In fact, all of the autosome copy number changes, with the exception of trisomy 21 (Down Syndrome), trisomy 18 (Edwards Syndrome) and trisomy 13 (Patau Syndrome), are not compatible with life after birth. Most babies born with trisomy 18 or 13 present several congenital abnormalities and die by age 1 [31, 32]. Recent mechanistic experiments on budding yeast have shown how aneuploidy directly affects gene expression at both the transcriptome and proteome levels and can generate significant phenotypic variation in a “dose dependent” fashion (the higher the degree of aneuploidy the higher the disruption of the phenotype) [33].

Yet, the presence of aneuploidy at the cellular level does not necessarily imply a disrupted phenotype at the organ level. High frequencies of aneuploidy have been reported in experimental conditions in the liver of knock-out mice after tyrosinemia-induced hepatic injury [34] and have been observed routinely through fluorescence in situ hybridization (FISH) in brain tissues of rodents and human [35], where mature aneuploid neurons were found to be functionally active and integrated into brain circuitry. This finding originally fueled speculation that aneuploidy might provide a selective advantage in these organs, but recent results obtained with single cell sequencing demonstrate that aneuploidy occurs much less frequently in the liver and brain than previously reported and is no more prevalent in these tissues than in skin [36]. In contrast, aneuploidy frequencies are significantly increased in organs affected by chronic degenerative diseases such as Alzheimer's disease, ataxia telangiectasia and liver cirrhosis [37, 38].

Because aneuploidy is well characterized and has multiple impacts on organismic health and development, it is an excellent biomarker for characterizing the reproductive toxicant and carcinogenic properties of chemicals. In the following sections we will review 1) the role of aneuploidy in cancer and reproductive toxicity; 2) the overlap among chemical classes between sperm genotoxicants, sperm aneuploidogens (substances inducing an altered number of whole chromosomes) and chemical carcinogens; 3) the different techniques available to assess aneuploidy in precancerous and malignant lesions and sperm; and 4) the experimental and epidemiological possibilities for integrating aneuploidy measurement into integrated chemical assessments.

## Aneuploidy in cancer and precancerous lesions

Aneuploidy, which includes both numerical and structural chromosomal abnormalities, is a hallmark of cancer [11]. It is a common characteristic of cancer and precancerous lesions, regardless of whether the causative agents are genotoxic, which directly damage the DNA, or non genotoxic carcinogens that may indirectly induce aneuploidy via different mechanisms, for example favoring higher replication rates in a tissue (as in the case of carcinogens with hormonal activity) and increasing the risk of errors during replication (in fact at each replication there is an intrinsic risk in human cells of developing de novo aneuploidy in 1:1000–1:10000 cells) [39–43]. By unbalancing the expression of thousands of genes [44] and proteins [45], aneuploidy disrupts the normal cell phenotype [46] and automatically destabilizes the karyotype, altering replication mechanisms [47] and catalyzing random aneuploidy events in single mitotic steps [48]. Cells from the same tumor are known to be clonal and in fact they share the majority of the karyotype (including clonal markers and chromosomal rearrangements), but every single cancer cell presents de novo numerical and/or structural chromosome rearrangements that are unique in each respective cell [49]. A recent analysis of 36,859 karyograms of 37 cancer types available in the Mitelman Database, the largest database of karyotypes of cancerous lesions from all sites maintained as part of the Cancer Genome Anatomy Project of the US National Cancer Institute (http://cgap.nci.nih.gov/Chromosomes/Mitelman), revealed that all the cancers were aneuploid; there was not a single case with a normal chromosome number (*n* = 46) that did not carry at least one structural or numerical aberration [50]. Indeed, karyotypic analysis of thousands of cancers has revealed the existence of karyotypic patterns, with aneuploidies that are recurrently found in several different cancer types, and others that are specific to individual tumors and tissues/organs of origin [51], but most aberrations appear to be non-specific and random [52], presenting individual, variant karyotypes, with characteristics each time resembling a new species [49, 53, 54].

A fundamental distinction between cancer and pre-cancerous cells is clonality: cancerous cells are clonal and aneuploid, which means all the cells from the primary and secondary lesions (metastasis) share the same ancestor and common markers [55, 56]. Clonality is routinely used as strict criteria for the pathological diagnosis of cancer, particularly, for the diagnosis of leukemia and lymphoma, and it can be assessed with different techniques (IHC, GSH, FISH, PCR) [55, 57]. In contrast, precancerous cells in dysplastic lesions are consistently aneuploid but not clonal. This may be better explained by investigating the relation between aneuploidy and field carcinogenesis. Field carcinogenesis (or cancerization) predicts that cancer occurs more often over dysplastic precancerous lesions induced by carcinogens [58], a concept originally proposed by Slaughter in 1953 for oral cancer [59] that now applies to every epithelial site. This explains the preventive efficacy of early diagnosis and treatment of dysplastic local lesions arising in specific “fields”, such as PAP-test (cervix cancer) [60], dysplastic polyps (colon cancer) [61], Barrett esophagus [62], leukoplakia (oral cancer) [63], and dysplastic nevi (melanoma) [64]. Virtually all dysplastic lesions present higher rates of aneuploid cells and their degree of aneuploidy often correlates with the likelihood of malignant progression in breast [65], colon [66], lung [67, 68], prostate [69], melanoma [70] oral [71], esophagus [72], cervix cancer [73] and leukemia [74].

Sperm aneuploidy can also be considered a preneoplastic lesion (e.g., men with Down and Klinefelter syndromes present higher rates of testicular cancer) as shown by Skakkebaek et al. [75]. Different authors have proposed tetraploidy as the first telltale step of the characteristic aneuploidy cascade of carcinogenesis [76–78]. Cancers have been shown to arise in non-clonal aneuploid dysplastic lesions induced by carcinogens in hamsters, mice, and rats exposed to carcinogens. When a clonal expansion occurs in dysplastic lesions, it is more appropriate to use the term carcinoma in situ, because clonality is an unique characteristic of cancer (carcinoma) cells [79, 80].

## Carcinogens cause aneuploidy

Whereas in biology aneuploidy often refers to any chromosomal imbalance (any karyotype different from diploid for somatic cells or different from haploid in the case of germinal cells), many efforts have been attempted in toxicology to differentiate clastogens (substances inducing disruption or breakages of chromosomes, leading to sections of the chromosome being deleted, added, or rearranged), from aneuploidogens [81]. Both clastogens and aneuploidogens can lead to the formation of micronuclei, a small extra-nucleus containing a whole chromosome or a fragment of a chromosome [82]. Several techniques have been developed for distinguishing clastogens from aneuploidogens in micronuclei tests, for example through the presence of a centromere/kinetokore for whole chromosomes, which are absent in chromosome fragments [83]. The distinction between the two categories is interesting for mechanistic purposes, yet they do not preclude similar biological consequences. Both clastogens and aneuploidogens are more likely to be carcinogens [84, 85], many carcinogens are both clastogens and aneuploidogens (for example X-rays and benzene) [86, 87], and both aneuploidogens and clastogens are able to foster further chromosomal instability (both partial and whole chromosome loss and gain) [88, 89]. Overall mechanisms and the continuum of the effects seem directly proportional to the disruption of the chromosomes. The inclusive definition of aneuploidy provided by Dyer in 1970 seems most consistent with its biological effects, “one or more whole chromosomes (numerical chromosomal aneuploidy) absent from or in addition to the euploid complement, or having one or more chromosome segments (segmental or structural aneuploidy) absent from or in addition to the euploid complement.” [13]. In fact, Oshimura and Barret, adopting Dyer’s definition, further specified that “an aneuploidogen may or may not be a clastogen” and anticipated that “aneuploidy in the form of a partial or complete chromosome duplication represents one form of gene amplification” [27].

## Aneuploidy in sperm and reproductive toxicity

Aneuploidy involving at least a single chromosome causes developmental abnormalities in all species where this condition has been examined [12]. An abnormal chromosome complement is thought to contribute to more than 50 % of early losses [90]. Many new gene mutations seen in offspring [91–93] and several abnormalities in the numbers of the sex chromosomes [94] come from the father’s sperm. Sperm aneuploidy is associated with infertility, miscarriages, and congenital abnormalities [95–97]. The most common aneuploidy in humans at birth involves an abnormal number of X or Y chromosomes, [98]. Children with sex chromosomal abnormalities, characterized in Klinefelter and Turner syndromes, can have reproductive disorders, behavioral difficulties, and/or reduced intellectual capabilities compared to their siblings [99, 100]. While US specific data are not available, European data from consecutive birth studies report the incidence of Klinefelter syndrome is increasing. A significant increase in the incidence of XXY trisomies in newborns between 1967 and 1988 has been reported [101], but no increases in the incidence of XXX or XYY trisomies. XXY trisomies frequently arise from nondisjunction of the XY (paternal) bivalent during meiosis I, and no observed increase in XXX trisomy (predominately maternally-derived) was observed, suggesting that there may be underlying environmental causes affecting non-disjunction during spermatogenesis. Data from the European Surveillance and Congenital Abnormalities [100] registry show significant regional differences in the prevalence of sex chromosome trisomies reported between 2000 and 2005; prevalence was lowest in Poland (0.19 per 10,000 births) and highest in Switzerland (5.36 per 10,000 births) [100]. These differences (up to 25 fold) might also be explained in part by the variation across European registries in the availability of prenatal screening (ultrasound and antenatal screening for Down’s syndrome) as well as organizational and cultural factors [100].

Elevated rates of aneuploidy in sperm are correlated with higher frequencies of chromosomal abnormalities in preimplantation embryos [102, 103], lower fertilization and implantation rates [104], and higher miscarriage rates [105]. Increased disomy is associated with inferior semen parameters in multivariate adjusted models [106]. Aneuploidy occurs when meiosis is disrupted during gametogenesis. It is not known how genotoxicants interfere with the meiotic phase, but infertile men often have an impaired chromosome synapsis and an increased frequency of chromosomes that are missing a recombination site [99, 107]. These errors make the cells susceptible to meiotic arrest and production of aneuploid gametes. Altered recombination impacts nondisjunction; non-recombinant chromosomes are susceptible to nondisjunction due to reduced connections among homologous chromosome pairs [108]. Chemicals known to disrupt hormone signaling have been shown to affect mammalian recombination and germ cell aneuploidy [109], while other genotoxicants induce structural and numerical chromosomal aberrations that are not repaired by male or female DNA repair machinery during fertilization [110]. Changes in the endocrinologic environment of the testis affect the rate of meiotic segregation errors [111], and *p,p*’-DDE for example has been shown to impact calcium ion channels (CatSper) to affect Ca2+ increases which impact sperm capacitation, chemotaxis, hyperactivation, and acrosomal exocytosis [112]. Men exposed to genotoxic compounds have been found to have significantly higher frequencies of chromosomally abnormal sperm [113–115].

## Sperm aneuploidogens and carcinogens

Carcinogens can be developmental and reproductive toxicants, but these effects may or may not follow similar pathways. How many carcinogens are capable of producing sperm aneuploidy and how many sperm aneuploidogens are also carcinogens? A complete answer is elusive to date, mainly because of the lack of sperm aneuploidy testing in experimental models and epidemiological cancer cohorts, but some initial evidence has emerged. A recent review of the reproductive health of men with occupational exposures [116] listed seven substances known to affect sperm genetic integrity: phthalates [117], styrene [118], organophosphates [119], carbaryl [120], fenvalerate [121], lead [122, 123], and benzene [124]. Six of these seven are known or suspected carcinogens according to the Report on Carcinogens of the US National Toxicology Program [125] or are listed by the State of California (http://oehha.ca.gov/prop65.html) as chemicals known to cause cancer. For one chemical (fenvalerate), adequate carcinogenicity data are not available. Chemicals that can induce sperm aneuploidy appear more likely to be carcinogenic. On the other hand, carcinogens should also be screened as potential sperm aneuploidogens, although it should be expected that only some of the aneuploidogens that are carcinogenic would also pass the blood-testis barrier. Additionally, germinal reproductive check points may prevent, at least in part, chromosomal damage. This is suggested by the finding that rates of aneuploid lymphocytes induced by benzene are significantly higher than aneuploidy rates in sperm [126]. Sperm aneuploidogens are often not only aneugenic to sperm, but also to other organs [43, 126–128].

## Techniques available to assess aneuploidy in precancerous and malignant lesions and sperm

**Fig. 1:**
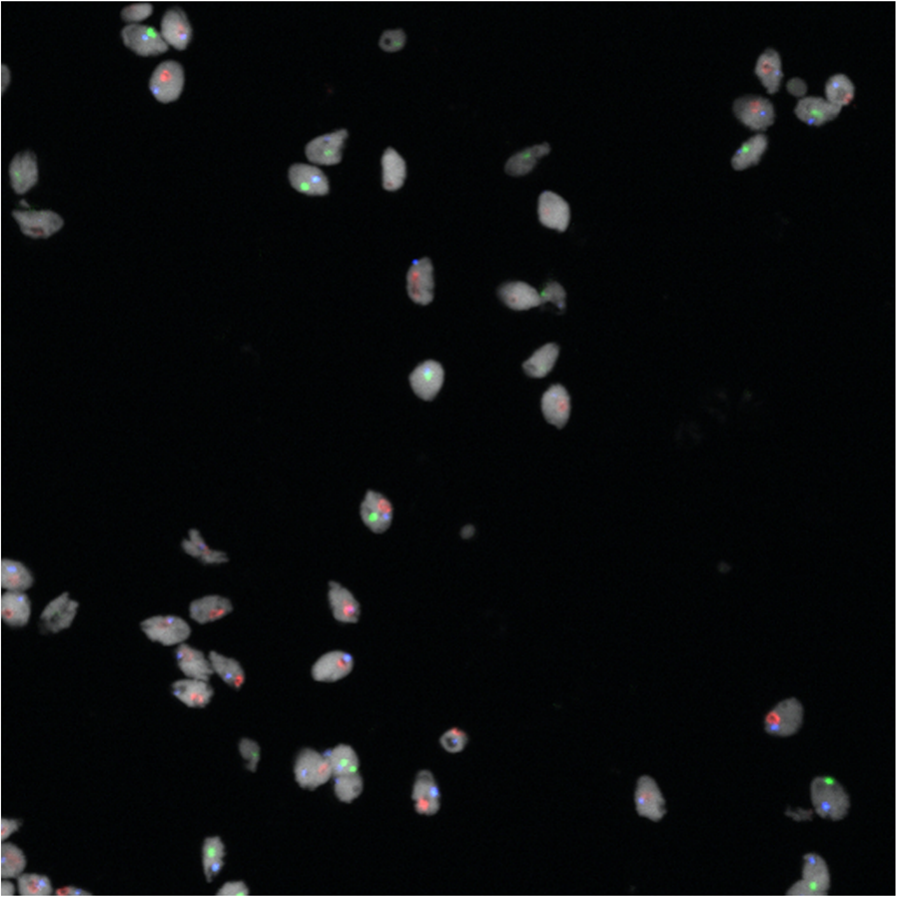
Sperm Fluorescence in situ hybridization results for chromosomes 18, X and Y. Full position image. Cells are displayed using a white for ToPro. Composite image of channels acquired for each of the four signals: SpectrumAqua (18), SpectrumGreen (X), SpectrumOrange (Y) and the nuclear stain

**Fig. 2:**
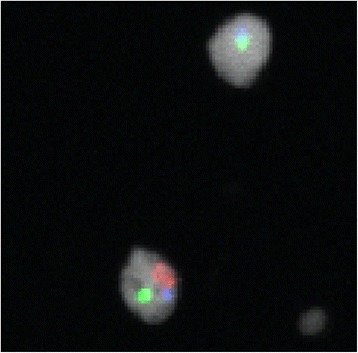
Sperm Fluorescence in situ hybridization results for chromosomes 18, X and Y. Snip image of the cells in white (cropped). Composite image of channels acquired for each of the four signals: SpectrumAqua (18), SpectrumGreen (X), SpectrumOrange (Y) and the nuclear stain

Aneuploidy, as an effect biomarker, is not a substitute for other fundamental indicators in cancer cells, such as genes and markers of proliferation (Ki-67), receptors that identify the tissue of origin of the lesion (IHC), or proteomic and genomic (and epigenomic) profiling of cancer cells. The main value of aneuploidy as an effect biomarker is that is a measurable characteristic of virtually any biological and pathological tissue: it can differentially diagnose pre-cancerous and cancerous lesions. In terms of its utility for sperm assessment, aneuploidy is informative well beyond the standard sperm quality indicators such as concentration, motility and morphology. Owing to techniques such as Laser Scanning Microscopy (Figs. 1 and 2), aneuploidy can be evaluated on the same slide, at a single cell level of resolution, together with other techniques and biomarkers such as IHC and Ki-67 for an optimal evidence based evaluation of malignancy, clonality, tissue of origin and replication rate of the lesion [129–131]. Progress in semiautomatic sperm scoring has advanced precision in determining sperm aneuploidy rates while saving on time and operator burden [132, 133] and is making human biomarker assessment more feasible for both cancer biology and molecular epidemiology laboratories [134].

## Future developments

Future advancements that will increase sensitivity in aneuploidy detection [135] include: improved interphase chromosome staining, with higher numbers of probes and aberrations tested on the same cell [136, 137] and the use of Quantum Dots that present higher fluorescence efficiency, lower photobleaching and vastly better photostability compared to organic fluorophores [138–140]. Another possible advancement will consist of the use of the specimens fixed in alcohol rather than formaldehyde [141]. Alcohol preserves DNA [142], RNA [143] and proteins [144] better than formaldehyde with comparable, if not superior, performance for molecular analysis, cytology and microscopy [145–148] and could dramatically lower the exposure of the operators to a known human carcinogen [149, 150].

Causes of oocyte aneuploidy are unknown, but it is the most common cause of pregnancy loss and birth defects [94]. Ovarian impacts are sometimes evaluated in toxicology studies, albeit less frequently than primary organs such as liver, lung, and brain. Oocyte aneuploidy might also be an interesting biomarker for chemical carcinogenicity or reproductive toxicity [151, 152]. Oocyte aneuploidy screening is likely also to advance in vitro fertilization techniques. In the controlled setting of the assisted reproduction clinic, aneuploidy in the embryo is the most frequently observed cause of failure to achieve a pregnancy. In this context, opportunities for more fully assessing oocyte aneuploidy are promising because women may be willing to donate remainder oocytes for epidemiologic and mechanistic studies [153–155].

## Conclusions

Aneuploidy is an excellent candidate as an evidence-based biomarker for reproductive toxicants and carcinogens. This condition is a cellular hallmark of all cancerous and precancerous lesions across a spectrum of cell types, and in sperm specifically, it is associated with infertility, miscarriages, and congenital abnormalities. Hence, sperm aneuploidy is of particular interest because this condition is associated with both increased risk of cancer and reproductive toxicity. Assessment of sperm aneuploidy induced by exposure to chemicals in both experimental models and epidemiological studies has proven effective and predictive of the risk to humans: of seven substances known to affect sperm genetic integrity [116], six are known or suspected carcinogens [125]. In light of these findings and considering that with the advent of automated chromosome counters and Laser Scanning Microscopy, sperm aneuploidy assessment has become much faster and reliable, it should be included to extend the evaluation of sperm aneuploidy in chemical hazard and risk assessment. Assessment of aneuploidy in sperm germinal cells should be integrated with current aneuploidy and chromosome imbalance assessments currently in place for somatic cells (such as comet and micronuclei assessments of peripheral lymphocytes) [156]. Assessments in both somatic and germ cells are needed to ensure that a comprehensive evaluation of carcinogenicity and reproductive toxicity is completed and one assessment cannot be substituted for the other, as they each provide independent information on chemical health impacts [157, 158].

## Abbreviations

Catsper: Cation channels of sperm
DNA: Deoxyribonucleic acid
FISH: Fluorescence in situ hybridization
GSH: Genome in situ hybridization
IHC: Immunohistochemistry
*p,p*’-DDE: Dichlorodiphenyldichloroethylene
PAP-test: Papanicolaou test
PCR: Polymerase chain reaction
RNA: Ribonucleic acid
